# Cancer cells increase endothelial cell tube formation and survival by activating the PI3K/Akt signalling pathway

**DOI:** 10.1186/s13046-017-0495-3

**Published:** 2017-02-07

**Authors:** Hao-Wei Cheng, Yi-Fang Chen, Jau-Min Wong, Chia-Wei Weng, Hsuan-Yu Chen, Sung-Liang Yu, Huei-Wen Chen, Ang Yuan, Jeremy J.W. Chen

**Affiliations:** 1 Institute of Biomedical Engineering, National Taiwan University, Taipei, Taiwan; 2 Graduate Institute of Clinical Dentistry, National Taiwan University, Taipei, Taiwan; 3 Department of Internal Medicine, National Taiwan University Hospital, Taipei, Taiwan; 4 Institute of Biomedical Sciences, National Chung-Hsing University, Taichung, Taiwan; 5 Institute of Statistical Science, Academia Sinica, Taipei, Taiwan; 6 Department of Clinical Laboratory Sciences and Medical Biotechnology, National Taiwan University, Taipei, Taiwan; 7 Graduate Institute of Toxicology, National Taiwan University, Taipei, Taiwan; 8 Department of Emergency Medicine, National Taiwan University Hospital, Taipei, Taiwan; 9 Department of Medical Research, China Medical University Hospital, China Medical University, Taichung, Taiwan

**Keywords:** Endothelial cell, Lung cancer, Angiogenesis, Apoptosis, PI3K/Akt

## Abstract

**Background:**

Angiogenesis is a hallmark of cancer and plays a critical role in lung cancer progression, which involves interactions between cancer cells, endothelial cells and the surrounding microenvironment. However, the gene expression profiles and the changes in the biological phenotype of vascular endothelial cells after interactions with lung cancer cells remain unclear.

**Methods:**

An indirect transwell co-culture system was used to survey the interaction between human umbilical vein endothelial cells (HUVECs) and human lung adenocarcinoma CL1-5 cells, as well as to investigate the morphological and molecular changes of HUVECs. The differentially expressed genes (DEGs) in HUVECs after co-culture with cancer cells were identified by microarray. Moreover, a publicly available microarray dataset of 293 non-small-cell lung cancer (NSCLC) patients was employed to evaluate the prognostic power of the gene signatures derived from HUVECs.

**Results:**

The interaction between HUVECs and lung cancer cells changes the morphology of HUVECs, causing them to have a mesenchymal-like morphology and alter their cytoskeleton organization. Furthermore, after co-culture with lung cancer cells, HUVECs showed increased cell motility and microvessel tube formation ability and a decreased apoptotic percentage. Transcriptomic profiling of HUVECs revealed that many survival-, apoptosis- and angiogenesis-related genes were differentially expressed after interactions with lung cancer cells. Further investigations showed that the PI3K/Akt signalling pathway and COX-2 are involved in endothelial tube formation under the stimulation of lung cancer cells. Moreover, Rac-1 activation might promote endothelial cell motility through the increased formation of lamellipodia and filopodia. The inhibitors of PI3K and COX-2 could reverse the increased tube formation and induce the apoptosis of HUVECs. In addition, the gene signatures derived from the DEGs in HUVECs could predict overall survival and disease-free survival in NSCLC patients and serve as an independent prognostic factor.

**Conclusions:**

In this study, we found that cancer cells can promote endothelial cell tube formation and survival, at least in part, through the PI3K/Akt signalling pathway and thus change the microenvironment to benefit tumour growth. The gene signatures from HUVECs are associated with the clinical outcome of NSCLC patients.

**Electronic supplementary material:**

The online version of this article (doi:10.1186/s13046-017-0495-3) contains supplementary material, which is available to authorized users.

## Background

The process by which new blood vessels sprout from pre-existing blood vessels is called angiogenesis. This step is normal and important in wound healing, cell growth and development [1]. However, angiogenesis is also a prerequisite for tumour progression and metastasis. Tumour angiogenesis provides oxygen and nutrients to cancer cells and also provides a route for cancer cell metastasis [2–4]. High tumour angiogenesis activity is associated with advanced tumour growth and metastasis in human cancers [5]. Moreover, the non-small-cell lung cancer (NSCLC) patients with higher microvessel densities in tumour tissues have a worse survival [6, 7].

The tumour microenvironment is composed of tumour cells, blood vessels, immune cells, fibroblasts and extracellular matrix (ECM) [8]. Many investigations have shown that the microenvironment plays a crucial role in tumour growth, metastasis and angiogenesis. Particularly, the proliferation and motility of endothelial cells are essential for microvessel sprout formation and angiogenesis, which correlates with cancer metastasis. The interaction between cancer, endothelial cells and other components of the microenvironment may have a reciprocal effect on angiogenesis, cancer cell proliferation and dissemination [9]. Vascular endothelial growth factor (VEGF) is a key regulator of angiogenesis. A previous meta-analysis showed that VEGF overexpression in tumour indicates an unfavourable prognosis in NSCLC patients [10]. Collagen VI, a major ECM protein in the microenvironment, can induce angiogenesis and promote tumour progression [11]. Cancer-associated fibroblasts promote angiogenesis, invasion and metastasis in many cancers [12, 13]. Tumour-associated macrophages stimulate tumour progression by inducing angiogenesis and suppressing adaptive immunity [14]. Our group also reported that M1-type macrophages could reduce tumour growth and angiogenesis in vitro and in vivo [15].

Dozens of studies have illustrated that the PI3K/Akt signalling pathway is critical in tumour growth, proliferation and survival. This makes PI3K/Akt and downstream signalling components suitable therapeutic targets [16, 17]. Moreover, several reports also show that PI3K and Akt play crucial roles in endothelial cell survival and angiogenesis and activated by VEGF, fibroblast growth factor (FGF) stimulation [18, 19]. However, the actual mechanisms that regulate tumour angiogenesis and cancer progression within the tumour microenvironment remain unclear. Previous data showed that glioma conditioned medium increased the proliferation, migration and tube formation of human brain endothelial cells via Roundabout4 (Robo4) down-regulation [20]. However, the mechanism behind the association between endothelial cells and lung cancer has not been defined. Lung cancer is one of the top ten causes of death in human malignancy and shows a poorer prognosis than other human cancers [21]. Therefore, the clarification of the molecular mechanism is essential for the discovery of new therapeutic reagents in the treatment of NSCLC.

In this study, we identified the differentially expressed genes of human endothelial cells after interaction with a human lung cancer cell line through an indirect co-culture system by microarray. A panel of genes involved in migration, tube formation and apoptosis were identified and validated. Inhibitors of the key activated genes (such as PI3K and COX-2) were applied to confirm the effect of these activated genes on the angiogenesis of endothelial cells after co-culture with lung cancer cells. These results may provide new therapeutic targets for anti-angiogenesis therapy for NSCLC in the future.

## Methods

### Cells

Human umbilical vein endothelial cells (HUVECs, pooled donors) were obtained from Lonza (Walkersville, MA) and maintained in Endothelial Basel Medium-2 (EBM-2) with supplements (Lonza). Replicated cultures were obtained by trypsinization and were used at passages < 6. The human lung adenocarcinoma cell line CL1-5 was established previously [22]. The cells were grown in RPMI 1640 medium (Thermo Fisher Scientific, Rockford, IL) supplemented with 10% FBS. All cell lines were kept at 37 °C in a humidified atmosphere containing 5% CO_**2**_. The Transwell Permeable Supports (Corning, Tewksbury, MA) with a 0.4 μm polycarbonate membrane were used in the co-culture model system to separate HUVECs and CL1-5 cells into the different compartments. One hour prior to co-culture, 5 × 10^4^ HUVECs in 500 μl EBM-2 were grown in 24-well plate with or without Matrigel coating (BD Biosciences, San Jose, CA). An equivalent number of CL1-5 cells in 200 μl EBM-2 were seeded into the top chamber of a transwell insert, which was then placed directly on top of the 24-well plate containing the HUVECs. When performing tube formation and TUNEL assay, HUVECs were seeded on Matrigel in 24-well plate. After incubation of overnight (mRNA and protein expression) or the indicated time (tube formation and TUNEL assay), HUVECs were harvested for further analysis as described below. For the motility assay, the 8 μm polycarbonate membrane insert was used in the co-culture system where HUVECs were in the upper and CL1-5 cells were in the lower compartment as described in the migration assay section.

### F-actin staining

For F-actin staining, cells were plated on 12-mm-diameter coverslips for 24 h, washed twice with phosphate-buffered saline (PBS) and fixed in 4% paraformaldehyde for 10 min at room temperature. After two additional washes with PBS, cells were permeabilized with 0.1% Triton X-100 for 2–5 min and washed again with PBS. Rhodamine conjugates of Phalloidin (Thermo Fisher Scientific) were used to stain F-actin. The cells were stained for 40 min at room temperature. The nuclei were stained with 4',6-diamidino-2-phenylindole (DAPI). The fluorescence images of F-actin and nuclei were visualized by confocal microscopy (ZEISS, Germany).

### Migration assay

The Transwell Permeable Support (Corning) with an 8-μm polycarbonate membrane insert was used in the cell migration model where HUVECs were allowed to migrate. Approximately 2 × 10^4^ HUVECs in 200 μL EBM-2 medium were loaded into each 24-well insert in triplicate with or without CL1-5 cells in the lower chamber at 37 °C in a 5% CO_2_ incubator. After approximately 20 h, the migrated cells were fixed with methanol, stained with Giemsa solution (Sigma, St. Louis, MO) and counted at 200x magnification under a light microscope.

### TUNEL assay

Detection and quantification of apoptosis were performed by the TUNEL reaction, using the *In Situ* Cell Death Detection Kit, Fluorescein (Roche Diagnostics, Indianapolis, IN). Cells were recovered from Matrigel by Cell Recovery Solution (Corning) after culture for 6, 12, 24 and 30 h, seeded onto slides by cytospin and stained following the standard protocol to label DNA strand breaks with fluorescein-dUTP. Propidium iodide (PI) was used to label all nuclei. The image data were analysed under a fluorescence microscope. Experiments were evaluated in triplicate, and 10 fields of view were quantified for each sample.

### Tube formation

Matrigel Basement Membrane Matrix (BD Biosciences) was diluted with EBM-2 medium and coated in 24-well plates at 37 °C for 1 h. Then, 5 × 10^4^ HUVECs were seeded alone or co-cultured with an equivalent number of CL1-5 cells in the EBM-2 medium on Matrigel. Co-cultured CL1-5 cells were seeded in transwells and incubated in the same well with HUVECs. The tube formation ability of HUVECs was measured at 1, 2, 6, 12 and 24 h with or without CL1-5 cells. In inhibitor experiments, HUVECs were treated with the PI3K inhibitor LY294002 (5 μM) and the COX-2 inhibitor celecoxib (10 μM) (Sigma) for 12 h and co-cultured with CL1-5 cells. After incubation, the number of tubes and nodes of the tubular structures was quantified.

### Real-time quantitative PCR

Total RNA was extracted from HUVECs, which were co-cultured with or without CL1-5 cells. First-strand cDNA for real-time quantitative PCR (QPCR) analysis was obtained from 5 μg of total RNA using a random primer and SuperScript III Reverse Transcriptase kit (Thermo Fisher Scientific) according to the manufacturer’s instructions. Reactions were detected by the SYBR Green approach (Thermo Fisher Scientific). Ten nanograms of cDNAs served as templates to detect gene expression. Experiments were performed three times in triplicate. Details of the specific primers designed for QPCR to determine relative levels of gene expression are shown in Table 1.

**Table 1:** Primer sequences used in real-time PCR experiments

Gene name	Primer sequence (5' → 3')
ACTN1	Forward: AACTGTCACTTGGCGGGCAGGG
	Reverse: AAGGGCATCAGCCAGGAGCAGAT
AKT3	Forward: CCTTCCAGACAAAAGACCGTTT
	Reverse: ATGTAGATAGTCCAAGGCAGAGACAA
CTNNB1	Forward: AGCTAAAATGGCAGTGCGTTTAG
	Reverse: ACTAGCCAGTATGATGAGCTTGCTT
CXCL8	Forward: TTGGCAGCCTTCCTGATTTC
	Reverse: AACTTCTCCACAACCCTCTGCA
ICAM1	Forward: CGATGACCATCTACAGCTTTCCGG
	Reverse: GCTGCTACCACAGTGATGATGACAA
ITGAV	Forward: CTTCCAATTGAGGA ATCACCAACT
	Reverse: CAATCCTGCTAGAACTGCTAAAATGA
ITGB3	Forward: CGACCGAAAAGAATTCGCTAAA
	Reverse: GGTACGTGATATTGGTGAAGGTAGAC
PIK3CA	Forward: AACACTCAAAGAGTACCTTGTTCCAA
	Reverse: TAGCACCCTTTCGGCCTTTA
PIK3R1	Forward: GCGAGATGGCACTTTTCTTGT
	Reverse: TACTTCGCCGTCCACCACTAC
PIK3R3	Forward: GATGCCCTATTCGACAGAACTGA
	Reverse: TTGGAACTGCTGAAGTCATTGG
PTGS2	Forward: CCCTTGGGTGTCAAAGGTAA
	Reverse: GCCCTCGCTTATGATCTGTC
RAC1	Forward: AAGCTGACTCCCATCACCTATCCG
	Reverse: CGAGGGGCTGAGACATTTACAACA
VCAM1	Forward: GGGAAGATGGTCGTGATCCTT
	Reverse: TCTGGGGTGGTCTCGATTTTA

### Western blot

All experiments were performed as previously described [23]. After transfer to nitrocellulose membranes, the following primary antibodies were used: α-actinin (Merck Millipore, Billerica, MA), β-catenin (SANTA CRUZ BIOTECHNOLOGY, Dallas, Texas), Akt (Cell Signaling Technology, Beverly, MA), phospho-Akt (Ser473) (Cell Signaling Technology), PI3K (SANTA CRUZ BIOTECHNOLOGY), phospho-PI3K p85 (Tyr458)/p55 (Tyr199) (Cell Signaling Technology), PARP (Cell Signaling Technology) and Caspase 3 (Cell Signaling Technology). Chemiluminescent signals were detected by the Fujifilm LAS-3000 system (Fujifilm, Tokyo, Japan), and β-actin and α-tubulin (Sigma) (Merck Millipore) were used as the loading control. To determine the Rac-1 activity, the Active Rac1 Pull-Down and Detection Kit was used, according to the manufacturer’s protocol (Thermo Fisher Scientific).

### Microarray analysis

The mRNA profiles of HUVECs co-cultured with or without CL1-5 cells were analysed using the Affymetrix Human Genome U133 Plus 2.0 GeneChip according to the manufacturer’s protocols (Santa Clara, CA) by the National Taiwan University Microarray Core Facility for Genomic Medicine. The raw data were analysed by GeneChip Operating software (GCOS). Pathway analyses of the differentially expressed genes were performed using the DAVID programme [24].

### Statistical analysis

The data were presented as the means ± standard deviations, and the significance of differences was analysed using Student’s t-test. All experiments were performed in triplicate. To evaluate the prognostic ability of the selected candidate genes, we examined their association with clinical data using a published microarray dataset GSE30219 [25]. The intensity values of the probes from expression profile of GSE30219 were first rescaled using a quantile normalization method, and then log-transformed to a base-2 scale. The prognostic test was performed as previously described [15]. Briefly, the differentially expressed genes were employed to test its association with overall survival and disease-free survival by univariate Cox proportional hazard regression analysis. For those genes with a significant Cox regression coefficient, a risk score method was used to calculate the signature and construct the risk score function. The risk score function was a linear combination of gene expression weighted by the regression coefficient from Cox regression. The median risk score was used as the cut-off point for patient classification. Kaplan-Meier method was used to estimate survival curve and difference between curves was evaluated by log-rank test. Multivariate Cox proportional hazards regression analysis was employed to evaluate independent prognostic factors, and age, gender and stage were used as covariates. All statistical tests were two-tailed and *P* < 0.05 was considered statistically significant.

## Results

### The phenotype of HUVECs changed after co-culture with CL1-5 cells

To clarify the interaction between endothelial cells and cancer cells, we established a co-culture system using the Corning 0.4 μm polycarbonate membrane. In this model, HUVECs were seeded in the lower chamber and CL1-5 lung cancer cells were seeded in the upper compartment. This system was labelled as HUVEC/CL1-5 in the figures, and HUVECs without cancer cells were labelled as HUVEC.

**Fig. 1:**
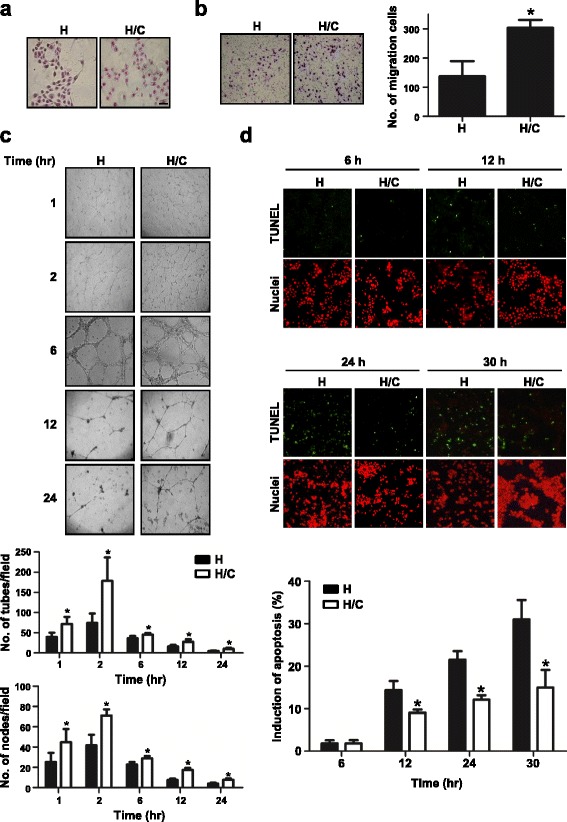
Phenotype changes of HUVECs after co-culture with CL1-5 cells. **a** Cell morphology of HUVECs co-cultured with or without CL1-5 cells, as determined by Giemsa Staining. Scale: 50 μm. **b** Migration ability and (**d**) apoptotic percentage of HUVECs after co-culture with CL1-5 cells, as measured by transwell and TUNEL assays, respectively. *Green:* TUNEL-positive nuclei. *Red:* all nuclei. *P < 0.05 compared with HUVECs only. **c** Phase contrast micrographs of the capillary-like tubular structures of HUVECs on Matrigel when cultured with or without CL1-5 cells for 1, 2, 6, 12, and 24 h. Bar graphs revealed the tube (*upper panel*) and node (*lower panel*) numbers (*P < 0.05). The data are presented as the mean ± SD. Experiments were performed in triplicate. Magnification, x100. H: HUVECs alone; H/C: HUVECs co-cultured with CL1-5 cells

We used the phenotypic changes of HUVECs to evaluate the effects of lung cancer cells on angiogenesis in the co-culture system. The morphology of HUVECs alone revealed a fusiform cell shape and formed a tight cluster. In contrast, after co-culture with CL1-5 cells, the HUVECs elongated, spread and lost contact with each other, as shown in Fig. 1a.

Angiogenesis is a multi-step process that includes proliferation, migration and tube formation of endothelial cells. Endothelial cells migrate along chemoattractants, which are secreted in the microenvironment. Here, we used the transwell migration assay to create the chemical gradient by putting cancer cells in the lower chamber. The data also showed that the migration capacity of HUVECs increased significantly (2.2-fold) when co-cultured with CL1-5 cells compared with HUVECs alone (*P* < 0.05) (Fig. 1b).

To evaluate the ability to undergo angiogenesis, the tube formation experiment is the key method used in vitro. When HUVECs were seeded on Matrigel, they gradually formed capillary-like tubular structures, and the capillary-like tubes connected to each other created a mesh-like structure on the gel. The capillary-like tubular structure was formed more densely, reached a peak at 2 h and finally disappeared in a time-dependent manner. Quantitative analysis of capillary-like tubular structures showed that the number of tubes and nodes significantly increased when HUVECs were co-cultured with CL1-5 cells at each time point (*P* < 0.05) (Fig. 1c). It appeared that the angiogenic ability of HUVECs increased after co-culture with cancer cells. On the other hand, the viability of endothelial cells is also critical in tumour angiogenesis [26]. As shown in Fig. 1c, the tubular structures decreased and the cell death increased as time went by. To further investigate whether the viability of HUVECs was altered by cancer cells, TUNEL assay was performed at 6, 12, 24 and 30 h after seeding HUVECs on Matrigel-coated plate. HUVECs were recovered from Matrigel by Cell Recovery Solution and estimated the apoptotic percentage. The percentages of apoptotic HUVECs were low at 6 h and showed no difference between co-culture and non-co-culture groups. However, the apoptotic percentage of HUVECs alone was increased 1.5 ~ 2-fold higher than that of HUVECs co-culture with CL1-5 cells and in a time dependent manner after 12, 24 and 30 h of tube formation (*P* < 0.05) (Fig. 1d). This indicated that co-culture with cancer cells would decrease the apoptotic percentage of endothelial cells.

### Gene expression and protein level changes in HUVECs after co-culture with CL1-5 cells

As shown in Fig. 1, many phenotypes of HUVECs changed when co-cultured with cancer cells. To identify the specific molecular mechanism, we used an Affymetrix microarray to identify the gene expression profile. We found that ~7000 genes were up-regulated and down-regulated over 2.5-fold in HUVECs co-cultured with CL1-5 cells compared to HUVECs alone. The differentially expressed genes were subjected to pathway analysis using DAVID. The top 10 significant pathways are listed in Additional file 1: Table S1. These functional pathways provided us with useful hints to confirm the molecular changes of HUVECs altered by cancer cells.

**Fig. 2:**
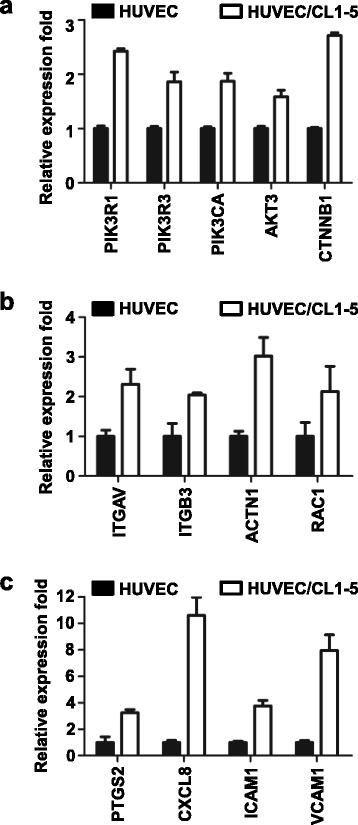
Increased expression of survival-, migration- and angiogenesis-related genes in HUVECs after co-culture with CL1-5 cells. The mRNA expression levels of the selected genes from microarray analysis were detected by QPCR. The fold changes of survival-related genes (**a**), migration-related genes (**b**) and angiogenesis-related genes (**c**) in HUVECs co-cultured with CL1-5 cells were calculated relative to the HUVECs alone group and normalized to internal control TBP. The data are presented as the mean ± SD. Experiments were performed in triplicate. Gene name versus protein name: CTNNB1 vs. β–catenin; ITGAV vs. integrin alpha 3; ITGB3 vs. integrin beta 3; ACTN1 vs. α-actinin; RAC1 vs. Rac-1; PTGS2 vs. COX-2; CXCL8 vs. IL-8

**Fig. 3:**
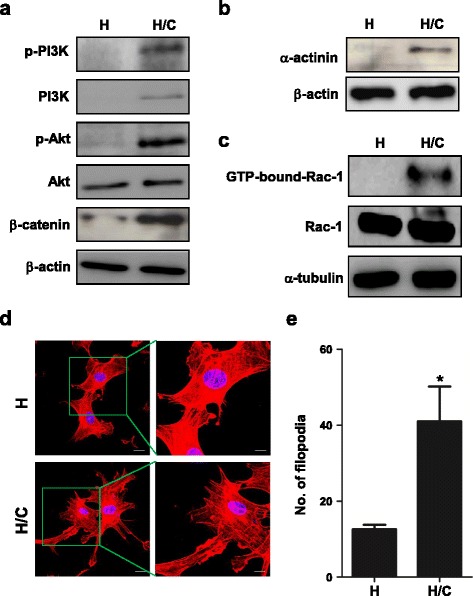
Protein changes and the F-actin distribution of HUVECs after co-culture with CL1-5 cells. **a**-**c** Immunoblots of the indicated proteins in HUVECs after interactions with CL1-5 cells for 24 h. The active Rac-1 was determined by the Active Rac1 Pull-Down and Detection Kit. β-actin or α-tubulin was used as the loading control. **d** Fluorescence images of lamellipodia and filopodia of HUVECs by F-actin staining. Scale: 20 μm and 10 μm (zoom in part). *Red*: F-actin. *Blue*: nuclear staining with DAPI. **e** The quantified number of filopodia of HUVECs co-cultured with CL1-5 cells (*P < 0.05) (*n* = 5). H: HUVECs alone; H/C: HUVECs co-cultured with CL1-5 cells. The data are presented as the mean ± SD. Experiments were performed in triplicate

According to pathway organization, we identified that PIK3CA, PIK3R3, AKT3, ITGAV, ITGB3 and RAC1 appeared in the top 2^nd^, 4^th^ and 5^th^ pathways (i.e., PI3K-Akt signalling pathway, Jak-STAT signalling pathway and RAP1 signalling pathway, respectively) and were correlated with the observed phenotypes. Although the changes in the expression of PIK3R1 and CTNNB1 were under 2.5-fold, these genes were also selected for further analysis due to their role in these three pathways. Moreover, based on a reference search, we further identified some genes enriched in the lower ranking of DAVID that predicted functional annotation or some genes with under a 2.5-fold change but related to the phenotype change of HUVECs, including ACTN1, PTGS2 (COX-2), CXCL8, VCAM1 and ICAM1. These candidate genes have been shown to be involved in survival, migration and angiogenesis. The expression of these genes was validated by QPCR, as shown in Fig. 2. In survival-related genes (Fig. 2a), all of the investigated genes were up-regulated in HUVECs with cancer cell co-culture. Among these, the mRNA levels of PIK3R1 and CTNNB1 were increased more than 2-fold, and those of PIK3R3, PIK3CA and AKT3 were increased 1.6- to 1.9-fold after co-culture with cancer cells. Nevertheless, the expression of these genes exhibited the same trend as the microarray data. In addition, all of the migration- and angiogenesis-related genes in HUVECs with CL1-5 co-culture were expressed 2-fold more than the genes in HUVECs alone (Fig. 2b, c). The comparison of the expression level between microarray and QPCR was summarized in Additional file 1: Table S2. The protein level and activity (phosphorylation status) were also confirmed by Western blot and showed a significant increase after co-culture with cancer cells (Fig. 3a, b).

### Rac-1, lamellipodia and filopodia were activated to promote cell motility in HUVECs after co-culture with CL1-5 cells

Based on our results, the migration ability of HUVECs was elevated after co-culture with cancer cells (Fig. 1b). In cell migration, Rho GTPases are important regulators of cell cytoskeleton dynamics [27]. Here, we showed that Rac-1 was bound by GTP after co-culture with CL1-5 cells (Fig. 3c). It revealed that Rac-1 was activated in HUVECs after co-culture with CL1-5, and this might contribute to the increasing motility of HUVECs after interaction with lung cancer cells.

Lamellipodia and filopodia are crucial in cell motility [28–30]. During cell movement, protrusions are stimulated at the leading edge of cells. We used rhodamine phalloidin to stain and label cytoskeleton F-actin in HUVECs and observed fluorescence images by confocal microscopy. The results showed that the cell margin of HUVECs alone group was smoother than that of the co-culture group. The cytoskeletal distribution of F-actin in HUVECs was rearranged after co-culture with CL1-5 cells. Filopodia stuck out of the lamellipodia (Fig. 3d). In addition, the number of filopodia increased in HUVECs after co-culture with CL1-5 cells (*P* < 0.05) (Fig. 3e).

### PI3K and COX-2 inhibitors attenuated the capillary-like tubular formation on Matrigel and promoted the apoptosis of HUVECs after co-culture with CL1-5 cells

**Fig. 4:**
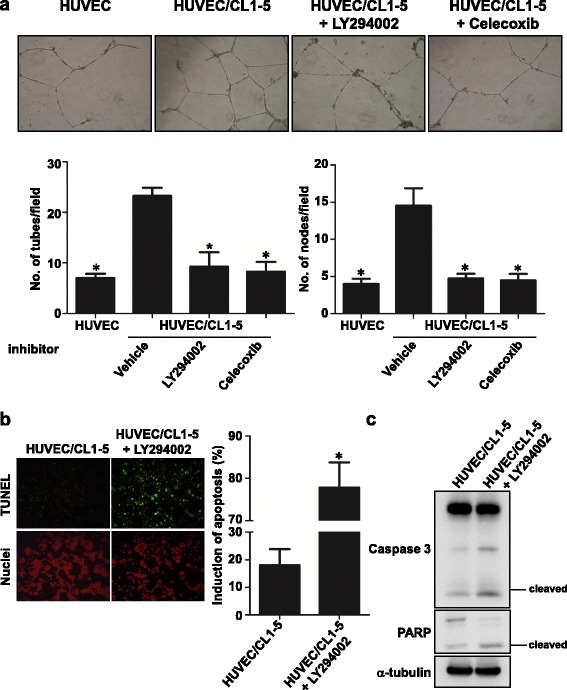
PI3K and COX-2 involved phenotype changes of HUVECs. **a** Phase contrast micrographs of capillary-like tubular structures on Matrigel. Magnification, x100. Graph bars showed the tube (*left panel*) and node (*right panel*) numbers of HUVECs treated with LY294002 or celecoxib in a co-culture system (*P < 0.05). **b** Apoptotic percentage of HUVECs with LY294002 treatment in the co-culture system as measured by TUNEL assay. *Green*: TUNEL-positive nuclei. *Red*: all nuclei. Bar graphs revealed the apoptotic percentage of HUVECs with LY294002 treatment after co-culture with CL1-5 cells (*P < 0.05). The data are presented as the mean ± SD. Experiments were performed in triplicate. **c** Immunoblot of cleaved Caspase 3 and PARP of HUVECs; α-tubulin was used as the loading control

In previous data, PI3K and COX-2 expression were significantly up-regulated in HUVECs after co-culture with CL1-5 cells (Figs. 2 and 3a). These proteins were reported to be involved in cell survival, migration and angiogenesis. Therefore, we chose inhibitors of PI3K (LY294002) and COX-2 (celecoxib) to clarify their effects on the capillary-like tubular formation capacity and cell viability of HUVECs after co-culture with CL1-5 cells. After being treated with 5 μM LY294002 and 10 μM celecoxib in HUVECs of the co-culture system, the capillary-like tubular structure on Matrigel was reduced significantly at 12 h compared with that of HUVEC/CL1-5 without inhibitor treatment. The number of tubes and nodes were also decreased in HUVEC/CL1-5 with treatment with LY294002 and celecoxib compared to the non-treated control (*P* < 0.05) (Fig. 4a). Furthermore, the apoptotic percentage increased from 18% to 78% compared with the non-treated control after LY294002 treatment by TUNEL staining assay (*P* < 0.05) (Fig. 4b). This apoptosis process involved Caspase 3 and PARP cleavage (Fig. 4c). These data revealed that PI3K and COX-2 were important in endothelial cell angiogenesis and survival. Manipulating PI3K and COX-2 could reverse the phenotypic changes of HUVECs after co-culture with lung cancer cells.

### Cancer cell-stimulated gene signatures were associated with the clinical outcome of NSCLC patients

**Fig. 5:**
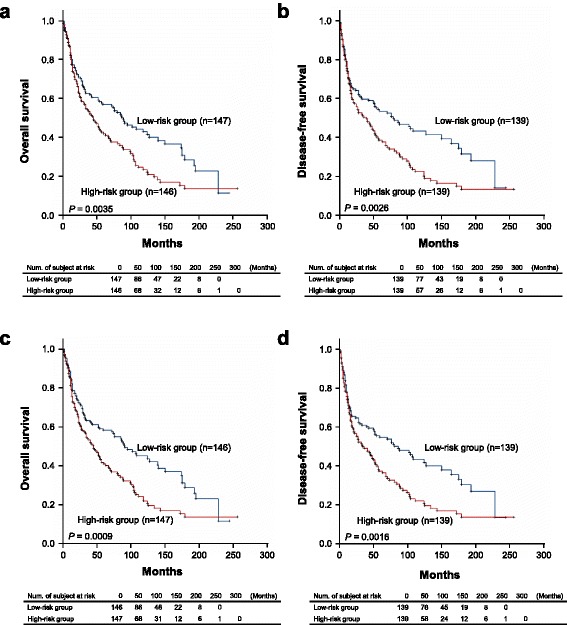
Kaplan–Meier estimates of NSCLC patient survival according to the stimulated endothelial cell gene signatures. **a**, **b** Kaplan-Meier estimates of overall survival (*n* = 293) (**a**) and disease-free survival (*n* = 278) (**b**) of the subjects who were categorized based on the 11-gene signature derived from the differentially expressed genes of HUVECs co-cultured with cancer cells. **c**, **d** Kaplan-Meier estimates of overall survival (*n* = 293) (**c**) and disease-free survival (*n* = 278) (**d**) of the subjects who were categorized based on the 5-gene signature derived from the differentially expressed genes of HUVECs co-cultured with cancer cells. The datasets were obtained from GSE30219. The survival curve was estimated by the Kaplan-Meier method, and the log-rank test was performed to test the difference between the survival curves

The data mentioned above indicated that many genes in endothelial cells were up-regulated after co-culture with cancer cells and correlated with angiogenesis, migration and anti-apoptosis phenotypes. Generally, the tumour specimen may contain not only endothelial cells but also surrounding tissue when the tumour section was dissected. Thus, we sought to determine whether these up-regulated gene sets could be employed to predict patient outcome. Based on microarray screening and QPCR validation (Fig. 2, Additional file 1: Table S2), 13 genes were used to construct the prognostic gene signatures. In these genes, two predicted the opposite clinical outcome compared to our results (ITGB3 and PIK3R1) and thus were not included in signature calculation. Using the remaining 11 genes that were differentially expressed after co-culture with cancer cells, we identified the gene signatures to predict the overall survival and disease-free survival of NSCLC patients. The 11-gene signature (12 probes from 11 genes) was identified based on the patient cohorts that had been published previously [25]. Detailed information on the probes, genes and risk score formula for each gene signature is described in Additional file 1: Table S3. Patients with a high expression gene signature exhibited shorter median overall survival and disease-free survival than patients with a low expression gene signature (*P* = 0.0035 and *P* = 0.0026, respectively; log rank test; Fig. 5a, b). Furthermore, we selected the hub genes or more upstream genes to narrow down the gene number, which may be more applicable in clinical testing. The 5-gene signature (6 probes from 5 genes) could more significantly predict overall survival and disease-free survival (*P* = 0.0009 and *P* = 0.0016, respectively; log rank test; Fig. 5c, d). A multivariate Cox proportional hazards regression with covariates of gender, age and stage was used to evaluate the prognostic independence of gene signatures in the published cohort (*n* = 293 in overall survival prediction; *n* = 278 in disease-free survival prediction). Except for the 11-gene set in disease-free survival, which was at the borderline but still had a trend (*P* = 0.0521), the gene signatures were significant after considering the effects of covariates (Table 2). The results indicated that the gene signatures from cancer cell-stimulated endothelial cells were correlated with extended overall survival and disease-free survival in this published cohort.

**Table 2:** Multivariate Cox regression analysis of two gene set signatures for the overall survival of patients with NSCLC

Variable	Hazard ratio	95% HR C.I.		*p*-value
11-gene set, overall survival				
Median of risk score	1.425	1.073	1.892	0.0143
Gender	1.460	0.926	2.300	0.1031
Age	1.037	1.022	1.052	<0.0001
Stage (0.1.2 vs. 3.4)	2.990	2.185	4.092	<0.0001
11-gene set, disease-free survival				
Median of risk score	1.341	0.997	1.803	0.0521
Gender	1.485	0.930	2.371	0.0974
Age	1.036	1.020	1.051	<0.0001
Stage (0.1.2 vs. 3.4)	2.941	2.116	4.087	<0.0001
5-gene set, overall survival				
Median of risk score	1.414	1.063	1.882	0.0172
Gender	1.455	0.923	2.293	0.1060
Age	1.037	1.022	1.052	<0.0001
Stage (0.1.2 vs. 3.4)	2.922	2.134	3.999	<0.0001
5-gene set, disease-free survival				
Median of risk score	1.356	1.009	1.823	0.0435
Gender	1.489	0.933	2.375	0.0950
Age	1.036	1.021	1.051	<0.0001
Stage (0.1.2 vs. 3.4)	2.903	2.087	4.037	<0.0001

## Discussion

The tumour microenvironment is a complex network composed of ECM, signalling molecules and different types of stromal cells, including infiltrating immune cells, cancer-associated fibroblasts, endothelial cells and pericytes [31]. They all play their own role to support tumour growth and restrict drugs from targeting the tumour centre. During tumour progression, malignant cells could also affect the microenvironment in many ways to promote immune tolerance and angiogenesis and sustain proliferative signalling. This influence makes for favourable surroundings and benefits tumour survival, proliferation and metastasis in cancer development [32]. In the present study, we found that the interaction between endothelial and lung cancer cells changed the HUVECs into a mesenchymal-like morphology, decreased the apoptotic percentage and increased the migration and tube forming ability of HUVECs. Using microarrays and the DAVID bioinformatics database, we identified several genes and signal transduction pathways affected in HUVECs after co-culture with CL1-5 cells. These differentially expressed genes were in accordance with the biological phenotype changes of HUVECs. Manipulating PI3K and COX-2 activity by inhibitors could reverse the tube formation ability and apoptotic resistance of HUVECs. These results could help to identify the feasible therapeutic target candidates for lung cancer anti-angiogenesis therapy in the future.

Angiogenesis is crucial in normal physiology. However, the imbalance of tumour angiogenesis activity is correlated with malignant tumour growth and metastasis in human cancers. To target angiogenesis in cancer therapy, the characterization of tumour-derived endothelial cells (TEC) from normal endothelial cells (NEC) is important. It has been shown that human hepatocellular carcinoma-derived endothelial cells showed increased proliferation, apoptosis resistance, migration and tube formation ability compared with NEC [33]. A previous study also showed that A549 conditioned medium (CM) increased HUVECs cell survival and wound healing migration ability and decreased apoptosis via Akt activation. Knockdown of Akt or treatment with the PI3K inhibitor wortmannin blocked A549 CM-induced cell survival and the migration ability of HUVECs [34]. Although cancer cells do not contact with endothelial cells in the Transwell or CM co-culture system, cancer cells can produce many growth factors and cytokines, such as VEGF, basic fibroblast growth factor (bFGF) and IL-8, in the medium to promote angiogenesis. VEGF is the most potent angiogenic factor that can stimulate endothelial cell proliferation and migration and decrease apoptosis via VEGF-VEGF receptor 2 signalling pathway. It also induces the adhesion molecules, ICAM1 and VCAM1, expression. In addition, bFGF not only modulates the expression of integrins but also stimulates VEGF secretion [35–37]. Interestingly, our data showed that some of the above molecules are also upregulated in HUVECs co-cultured with lung cancer cells.

In the present study, we also found that interaction with lung cancer cells changed the HUVECs into a longer, mesenchymal-like morphology and increased the migration, anti-apoptosis and tube formation ability (Fig. 1). However, the proliferation rate did not change in our system (data not shown). Furthermore, we identified that not only Akt but PI3K and Rac-1 were activated in HUVECs after co-culture with CL1-5 cells (Fig. 3a, c). In the previous report, the authors only investigated the apoptosis resistance of NEC or A549 CM-treated HUVECs by inoculating endothelial cells in culture plates in serum starvation. Our data provided further evidence that the interaction with cancer cells decreased the apoptosis percentage of HUVECs on Matrigel, which is more similar to ECM than the culture dish only. Furthermore, the tube formation assay is closer to the process of angiogenesis in physiological conditions than the wound healing assay to mimic vessel formation.

PI3K/Akt signalling elicits many downstream signalling pathways, which are related to cell growth, cell survival, cell cycle, apoptosis, cell motility, glucose metabolism and angiogenesis [38]. Our study revealed that the interaction with cancer cells increased the PI3K and Akt mRNA levels of HUVECs, as well as their activities. This activation turned on the downstream expression of genes such as β–catenin and stimulated tube formation and apoptosis induction (Figs. 1c, d and 2a). Previous studies reported that β3 integrin induces calpain-dependent integrin cluster formation, triggers Rac-1 activation and ultimately leads to the formation of Rac-1-induced focal complexes. Rac-1 is an important regulator of actin polymerization at the cell’s leading edge. Knockdown of α-actinin impairs Rac-1 induced dorsal stress fibre formation [39, 40]. Here, we showed that the mRNA of integrin alpha V, integrin beta 3, α-actinin and Rac-1, as well as the protein level of α-actinin and active Rac-1 of HUVECs, were elevated after interaction with cancer cells (Figs. 2b and 3b, c). These changes resulted in filopodia and lamellipodia formation and the increase of migration (Figs. 1b and 3d, e). COX-2, IL-8, ICAM1 and VCAM1 are angiogenesis-related genes [37]. IL-8 induces ICAM1, VCAM1 and COX-2 expression [36]. Celecoxib decreases ICAM1 and VCAM1 expression in HUVECs and inhibits HT29 cells’ adhesion to endothelial cells [41]. Our data showed that the interaction of cancer and endothelial cells increases COX-2, IL-8, ICAM1 and VCAM1 mRNA levels of HUVECs (Fig. 2c). The inhibition of COX-2 by celecoxib prevented tube formation when co-cultured with cancer cells (Fig. 4a).

COX-2 is overexpressed in many cancers and is associated with angiogenesis through the Rac/Cdc42 and PI3K-Ras signalling pathway [42–44]. It has been shown that COX-2 cross-talks with PI3K/Akt in epithelial ovarian cancer. The inhibition of COX-2 by aspirin and COX-2 siRNA decreased Akt phosphorylation and the downstream signalling pathway [45]. In angiogenesis, PI3K/Akt activation induces VEGF production in cancer cells. Once VEGF binds to the receptor, this binding also turns on the PI3K signalling pathway in endothelial cells and causes actin reorganization and cell migration and finally increases angiogenesis. Here, we also showed that the mRNA level of COX-2 and PI3K and the quantity of active PI3K protein was increased in HUVECs after co-culture with cancer cells (Figs. 2 and 3a). When we treated HUVECs with PI3K and COX-2 inhibitors in the co-culture system, the angiogenesis ability of HUVECs was reduced (Fig. 4). This indicated that cancer cells could influence the endothelial cell phenotype via the PI3K and COX-2 pathways, at least in our experimental conditions. Taken together, COX-2 and PI3K could not only be activated in cancer cells but also in endothelial cells to induce angiogenesis through the interaction of cancer and endothelial cells. This cross-talk creates a microenvironment beneficial for tumour growth and finally makes tumour cells malignant.

In clinical outcome analysis, tumour samples are typically collected to determine the gene expression profiles and survival information. However, the tumour mass was composed not only of cancer cells but also of other surrounding cell types, including endothelial cells, immune cells and stroma cells, which exist in the tumour microenvironment [8]. Thus, we tried to use the differentially expressed genes in HUVECs after co-culture with cancer cells to predict the clinical outcome. Our data indicated that these cancer cell-stimulated genes could estimate patients’ overall survival and disease-free survival (Fig. 5). This result suggested that these genes were also important in cancer cells. The cause may be the cross-talk between cancer and the microenvironment as previously described. Furthermore, we also compared the predictive power between our HUVECs derived gene signatures and the published prognostic gene signatures, 8-gene [46] and 7-gene [47], using the same dataset GSE30219 as ours. To perform the comparisons appropriately, the 8- and 7-gene signatures were employed to construct the risk score functions according to our method and then subjected to overall survival analyses. Interestingly, the re-calculated log-rank tests were more significant (*P* < 0.0001 and *P* = 0.0045, respectively; Additional file 1: Figure S1a and S1b) compared to the original studies (*P* = 0.000513 and *P* = 0.0071, respectively) in Kaplan-Meier estimates. The results also showed that both our gene signatures could predict patient prognosis as well as the published 8-gene signature did and better than the 7-gene signature in early-stage lung adenocarcinoma (*P* < 0.0001 and *P* = 0.0029, respectively; log rank test; Additional file 1: Figure S1c and S1d). It is worth mentioning that our 5-gene signature has the fewest genes among the signatures compared here. Therefore, taking the gene expression profile of endothelial cells into account may improve the prognostic prediction.

## Conclusions

The growth of new blood vessels is an essential process for tumour progression, which makes angiogenesis a favourable target in anti-cancer therapy. Among the factors that promote angiogenesis, the tumour microenvironment plays a pivotal role, as described in this study and others [33]. It is reasonable to propose that breaking down the interaction or communication between cancer cells and endothelial cells is an ideal approach for the treatment of lung cancer patients. In this study, we found that COX-2 and PI3K inhibitors could reverse the angiogenic phenotype of endothelial cells. Actually, several PI3K inhibitors such as BKM120 and XL147 are under clinical development as a single agent therapy or in combination with other drugs in NSCLC and other solid tumours [48–51]. Therefore, the application of antitumour drugs with COX-2 and/or PI3K inhibition activity or in combination with the indicated inhibitors can be a feasible strategy for developing cancer therapies in the future.

## Additional file

Additional file 1: Table S1. Pathway analysis of the differentially expressed genes in HUVECs with cancer cell co-culture. **Table S2.** The differentially expressed gene changes in microarray data and QPCR validation. **Table S3.** Probes and gene list of cancer cell-stimulated gene signatures derived from endothelial cells. **Figure S1.** Comparison of the endothelial cell derived gene signatures and the published prognostic gene signatures in the Kaplan–Meier estimates of NSCLC patient survival. (DOCX 178 kb)

## Abbreviations

CM: Conditioned medium
DEGs: Differentially expressed genes
EBM-2: Endothelial basel medium-2
ECM: Extracellular matrix
FGF: Fibroblast growth factor
GCOS: GeneChip operating software
HUVECs: Human umbilical vein endothelial cells
NEC: Normal endothelial cells
NSCLC: Non-small-cell lung cancer
PBS: Phosphate-buffered saline
PI: Propidium iodide
Robo4: Roundabout4
TEC: Tumour-derived endothelial cells
VEGF: Vascular endothelial growth factor

## References

[1] Carmeliet P (2003). Angiogenesis in health and disease. Nat Med.

[2] Hoeben A, Landuyt B, Highley MS, Wildiers H, Van Oosterom AT, De Bruijn EA (2004). Vascular endothelial growth factor and angiogenesis. Pharmacol Rev.

[3] Folkman J (2002). Role of angiogenesis in tumor growth and metastasis. Semin Oncol.

[4] Weis SM, Cheresh DA (2011). Tumor angiogenesis: molecular pathways and therapeutic targets. Nat Med.

[5] Hicklin DJ, Ellis LM (2005). Role of the vascular endothelial growth factor pathway in tumor growth and angiogenesis. J Clin Oncol.

[6] Jusufovic E, Rijavec M, Keser D, Korosec P, Sodja E, Iljazovic E, Radojevic Z, Kosnik M (2012). let-7b and miR-126 are down-regulated in tumor tissue and correlate with microvessel density and survival outcomes in non--small--cell lung cancer. PLoS One.

[7] Kadota K, Huang CL, Liu D, Ueno M, Kushida Y, Haba R, Yokomise H (2008). The clinical significance of lymphangiogenesis and angiogenesis in non-small cell lung cancer patients. Eur J Cancer.

[8] Nyberg P, Salo T, Kalluri R (2008). Tumor microenvironment and angiogenesis. Front Biosci.

[9] Reymond N, d'Agua BB, Ridley AJ (2013). Crossing the endothelial barrier during metastasis. Nat Rev Cancer.

[10] Zheng CL, Qiu C, Shen MX, Qu X, Zhang TH, Zhang JH, Du JJ (2015). Prognostic impact of elevation of vascular endothelial growth factor family expression in patients with non-small cell lung cancer: an updated meta-analysis. Asian Pac J Cancer Prev.

[11] Chen P, Cescon M, Bonaldo P (2013). Collagen VI in cancer and its biological mechanisms. Trends Mol Med.

[12] Mao Y, Keller ET, Garfield DH, Shen K, Wang J (2013). Stromal cells in tumor microenvironment and breast cancer. Cancer Metastasis Rev.

[13] Shiga K, Hara M, Nagasaki T, Sato T, Takahashi H, Takeyama H (2015). Cancer-associated fibroblasts: their characteristics and their roles in tumor growth. Cancers (Basel).

[14] Mantovani A, Schioppa T, Porta C, Allavena P, Sica A (2006). Role of tumor-associated macrophages in tumor progression and invasion. Cancer Metastasis Rev.

[15] Yuan A, Hsiao YJ, Chen HY, Chen HW, Ho CC, Chen YY, Liu YC, Hong TH, Yu SL, Chen JJ, Yang PC (2015). Opposite effects of M1 and M2 macrophage subtypes on lung cancer progression. Sci Rep.

[16] Rodon J, Dienstmann R, Serra V, Tabernero J (2013). Development of PI3K inhibitors: lessons learned from early clinical trials. Nat Rev Clin Oncol.

[17] Thorpe LM, Yuzugullu H, Zhao JJ (2015). PI3K in cancer: divergent roles of isoforms, modes of activation and therapeutic targeting. Nat Rev Cancer.

[18] Gerber HP, McMurtrey A, Kowalski J, Yan M, Keyt BA, Dixit V, Ferrara N (1998). Vascular endothelial growth factor regulates endothelial cell survival through the phosphatidylinositol 3'-kinase/Akt signal transduction pathway. Requirement for Flk-1/KDR activation. J Biol Chem.

[19] Shiojima I, Walsh K (2002). Role of Akt signaling in vascular homeostasis and angiogenesis. Circ Res.

[20] Cai H, Xue Y, Li Z, Hu Y, Wang Z, Liu W, Li Z, Liu Y (2015). Roundabout4 suppresses glioma-induced endothelial cell proliferation, migration and tube formation in vitro by inhibiting VEGR2-mediated PI3K/AKT and FAK signaling pathways. Cell Physiol Biochem.

[21] Siegel RL, Miller KD, Jemal A (2016). Cancer statistics, 2016. CA Cancer J Clin.

[22] Chu YW, Yang PC, Yang SC, Shyu YC, Hendrix MJ, Wu R, Wu CW (1997). Selection of invasive and metastatic subpopulations from a human lung adenocarcinoma cell line. Am J Respir Cell Mol Biol.

[23] Chen CC, Chen HY, Su KY, Hong QS, Yan BS, Chen CH, Pan SH, Chang YL, Wang CJ, Hung PF (2014). Shisa3 is associated with prolonged survival through promoting beta-catenin degradation in lung cancer. Am J Respir Crit Care Med.

[24] da Huang W, Sherman BT, Lempicki RA (2009). Systematic and integrative analysis of large gene lists using DAVID bioinformatics resources. Nat Protoc.

[25] Rousseaux S, Debernardi A, Jacquiau B, Vitte AL, Vesin A, Nagy-Mignotte H, Moro-Sibilot D, Brichon PY, Lantuejoul S, Hainaut P (2013). Ectopic activation of germline and placental genes identifies aggressive metastasis-prone lung cancers. Sci Transl Med.

[26] Dimmeler S, Zeiher AM (2000). Endothelial cell apoptosis in angiogenesis and vessel regression. Circ Res.

[27] Heasman SJ, Ridley AJ (2008). Mammalian Rho GTPases: new insights into their functions from in vivo studies. Nat Rev Mol Cell Biol.

[28] Nobes CD, Hall A (1995). Rho, rac, and cdc42 GTPases regulate the assembly of multimolecular focal complexes associated with actin stress fibers, lamellipodia, and filopodia. Cell.

[29] Hall A (1998). Rho GTPases and the actin cytoskeleton. Science.

[30] Small JV, Stradal T, Vignal E, Rottner K (2002). The lamellipodium: where motility begins. Trends Cell Biol.

[31] Hanahan D, Coussens LM (2012). Accessories to the crime: functions of cells recruited to the tumor microenvironment. Cancer Cell.

[32] Liotta LA, Kohn EC (2001). The microenvironment of the tumour-host interface. Nature.

[33] Xiong YQ, Sun HC, Zhang W, Zhu XD, Zhuang PY, Zhang JB, Wang L, Wu WZ, Qin LX, Tang ZY (2009). Human hepatocellular carcinoma tumor-derived endothelial cells manifest increased angiogenesis capability and drug resistance compared with normal endothelial cells. Clin Cancer Res.

[34] Tu ML, Wang HQ, Chen LJ, Lu JC, Jiang F, Liang JH, Xu DG, Li DS (2009). Involvement of Akt1/protein kinase Balpha in tumor conditioned medium-induced endothelial cell migration and survival in vitro. J Cancer Res Clin Oncol.

[35] Shijubo N, Kojima H, Nagata M, Ohchi T, Suzuki A, Abe S, Sato N (2003). Tumor angiogenesis of non-small cell lung cancer. Microsc Res Tech.

[36] Manna SK, Ramesh GT (2005). Interleukin-8 induces nuclear transcription factor-kappaB through a TRAF6-dependent pathway. J Biol Chem.

[37] Sprague AH, Khalil RA (2009). Inflammatory cytokines in vascular dysfunction and vascular disease. Biochem Pharmacol.

[38] Liu P, Cheng H, Roberts TM, Zhao JJ (2009). Targeting the phosphoinositide 3-kinase pathway in cancer. Nat Rev Drug Discov.

[39] Bialkowska K, Kulkarni S, Du X, Goll DE, Saido TC, Fox JE (2000). Evidence that beta3 integrin-induced Rac activation involves the calpain-dependent formation of integrin clusters that are distinct from the focal complexes and focal adhesions that form as Rac and RhoA become active. J Cell Biol.

[40] Kovac B, Teo JL, Makela TP, Vallenius T (2013). Assembly of non-contractile dorsal stress fibers requires alpha-actinin-1 and Rac1 in migrating and spreading cells. J Cell Sci.

[41] Dianzani C, Brucato L, Gallicchio M, Rosa AC, Collino M, Fantozzi R (2008). Celecoxib modulates adhesion of HT29 colon cancer cells to vascular endothelial cells by inhibiting ICAM-1 and VCAM-1 expression. Br J Pharmacol.

[42] Catalano V, Turdo A, Di Franco S, Dieli F, Todaro M, Stassi G (2013). Tumor and its microenvironment: a synergistic interplay. Semin Cancer Biol.

[43] Salvado MD, Alfranca A, Haeggstrom JZ, Redondo JM (2012). Prostanoids in tumor angiogenesis: therapeutic intervention beyond COX-2. Trends Mol Med.

[44] Wang D, DuBois RN (2004). Cyclooxygenase 2-derived prostaglandin E2 regulates the angiogenic switch. Proc Natl Acad Sci U S A.

[45] Uddin S, Ahmed M, Hussain A, Assad L, Al-Dayel F, Bavi P, Al-Kuraya KS, Munkarah A (2010). Cyclooxygenase-2 inhibition inhibits PI3K/AKT kinase activity in epithelial ovarian cancer. Int J Cancer.

[46] Shahid M, Choi TG, Nguyen MN, Matondo A, Jo YH, Yoo JY, Nguyen NN, Yun HR, Kim J, Akter S (2016). An 8-gene signature for prediction of prognosis and chemoresponse in non-small cell lung cancer. Oncotarget.

[47] Krzystanek M, Moldvay J, Szuts D, Szallasi Z, Eklund AC (2016). A robust prognostic gene expression signature for early stage lung adenocarcinoma. Biomark Res.

[48] Bendell JC, Rodon J, Burris HA, de Jonge M, Verweij J, Birle D, Demanse D, De Buck SS, Ru QC, Peters M (2012). Phase I, dose-escalation study of BKM120, an oral pan-Class I PI3K inhibitor, in patients with advanced solid tumors. J Clin Oncol.

[49] Vansteenkiste JF, Canon JL, Braud FD, Grossi F, De Pas T, Gray JE, Su WC, Felip E, Yoshioka H, Gridelli C (2015). Safety and efficacy of buparlisib (BKM120) in patients with PI3K pathway-activated non-small cell lung cancer: results from the phase II BASALT-1 study. J Thorac Oncol.

[50] Shapiro GI, Rodon J, Bedell C, Kwak EL, Baselga J, Brana I, Pandya SS, Scheffold C, Laird AD, Nguyen LT (2014). Phase I safety, pharmacokinetic, and pharmacodynamic study of SAR245408 (XL147), an oral pan-class I PI3K inhibitor, in patients with advanced solid tumors. Clin Cancer Res.

[51] Massacesi C, Di Tomaso E, Urban P, Germa C, Quadt C, Trandafir L, Aimone P, Fretault N, Dharan B, Tavorath R, Hirawat S (2016). PI3K inhibitors as new cancer therapeutics: implications for clinical trial design. Onco Targets Ther.

