# Supplementary material for: Cancer cells increase endothelial cell tube formation and survival by activating the PI3K/Akt signalling pathway
Source: J Exp Clin Cancer Res. 2017 Feb 7;36:27. doi: 10.1186/s13046-017-0495-3 (PMC5296960; doi:10.1186/s13046-017-0495-3)
Supplement: Additional file 1: Table S1. — Pathway analysis of the differentially expressed genes in HUVECs with cancer cell co-culture. Table S2. The differentially expressed gene changes in microarray data and QPCR validation. Table S3. Probes and gene list of cancer cell-stimulated gene signatures derived from endothelial cells. Figure S1. Comparison of the endothelial cell derived gene signatures and the published prognostic gene signatures in the Kaplan–Meier estimates of NSCLC patient survival. (DOCX 178 kb) [file 13046_2017_495_MOESM1_ESM.docx]

**Additional file 1**

**Cancer cells increase endothelial cell tube formation and survival**

**by activating the PI3K/Akt signalling pathway**

Hao-Wei Cheng, Yi-Fang Chen, Jau-Min Wong, Chia-Wei Weng, Hsuan-Yu Chen, Sung-Liang Yu, Huei-Wen Chen, Ang Yuan, and Jeremy J.W. Chen

Correspondence: Associate Professor Ang Yuan, E-mail: [navyyuan@ntu.edu.tw](mailto:navyyuan@ntu.edu.tw) or Professor Jeremy J.W. Chen, E-mail: [jwchen@dragon.nchu.edu.tw](mailto:jwchen@dragon.nchu.edu.tw)

**Table S1.** **Pathway analysis of the differentially expressed genes in HUVECs with cancer cell co-culture.**

| **Significant Pathways** | **Total** | **Found** | **p-Value** |
| --- | --- | --- | --- |
| **Neuroactive ligand-receptor interaction** | 277 | 141 | 3.6E-10 |
| **PI3K-Akt signalling pathway** | 345 | 153 | 5.1E-6 |
| **Insulin secretion** | 85 | 46 | 9.1E-5 |
| **Jak-STAT signalling pathway** | 145 | 69 | 2.5E-4 |
| **Rap1 signalling pathway** | 210 | 94 | 2.8E-4 |
| **Aldosterone synthesis and secretion** | 81 | 43 | 2.8E-4 |
| **Cytokine-cytokine receptor interaction** | 229 | 101 | 3.1E-4 |
| **Calcium signalling pathway** | 175 | 80 | 3.8E-4 |
| **Glutamatergic synapse** | 114 | 56 | 4.0E-4 |
| **Cell adhesion molecules (CAMs)** | 142 | 67 | 4.3E-4 |

Note:

1. The differentially expressed genes were defined as the genes with a fold change greater than 2.5 between HUVECs with and without cancer cell co-culture.
2. The top 10 pathways were determined by the DAVID programme.

**Table S2. The differentially expressed gene changes in microarray data and QPCR validation.**

| **Array probe ID** | **Gene symbol** | **Array ratio^1^** | **QPCR fold change^2^** |
| --- | --- | --- | --- |
| **211579_at** | ITGB3 | 7.25 | 2.04 |
| **242876_at** | AKT3 | 5.63 | 1.58 |
| **237401_at** | ACTN1 | 5.62 | 3.02 |
| **202859_x_at** | CXCL8 | 5.44 | 10.59 |
| **211506_s_at** | CXCL8 | 4.82 | 10.59 |
| **241325_at** | PIK3R3 | 4.57 | 1.86 |
| **204369_at** | PIK3CA | 3.67 | 1.87 |
| **202351_at** | ITGAV | 3.40 | 2.31 |
| **203868_s_at** | VCAM1 | 2.92 | 7.93 |
| **1567457_at** | RAC1 | 2.54 | 2.13 |
| **212239_at** | PIK3R1 | 2.45 | 2.42 |
| **1554997_a_at** | PTGS2 | 1.82 | 3.25 |
| **202638_s_at** | ICAM1 | 1.65 | 3.76 |
| **201533_at** | CTNNB1 | 1.65 | 2.71 |

Note:

1. Array ratio = expression of co-cultured HUVECs/expression of HUVECs alone.
2. QPCR fold change = expression of co-cultured HUVECs/expression of HUVECs alone.

**Table S3. Probes and gene list of cancer cell-stimulated gene signatures derived from endothelial cells.**

| **Probe ID** | **Gene name** | **Cox coefficient** | | **Hazard ratio** | |
| --- | --- | --- | --- | --- | --- |
|  |  | **OS*** | **DFS*** | **OS** | **DFS** |
| **202351_at** | ITGAV | 0.54833 | 0.50411 | 1.730 | 1.656 |
| **204369_at** | PIK3CA | 1.08186 | 1.43882 | 2.950 | 4.216 |
| **241325_at** | PIK3R3 | 1.15214 | 1.24444 | 3.165 | 3.471 |
| **242876_at** | AKT3 | 0.91519 | 0.95833 | 2.497 | 2.607 |
| **1567457_at** | RAC1 | 0.44776 | 0.15455 | 1.565 | 1.167 |
| **237401_at** | ACTN1 | 0.12674 | 0.77845 | 1.135 | 2.178 |
| **203868_s_at** | VCAM1 | 0.35977 | 0.37022 | 1.433 | 1.448 |
| **202859_x_at** | CXCL8 | 0.74904 | 0.73972 | 2.115 | 2.095 |
| **211506_s_at** | CXCL8 | 0.53920 | 0.51848 | 1.715 | 1.679 |
| **201533_at** | CTNNB1 | 1.29538 | 0.85054 | 3.652 | 2.341 |
| **1554997_a_at** | PTGS2 | 0.07447 | 0.05834 | 1.077 | 1.060 |
| **202638_s_at** | ICAM1 | 0.01016 | -0.03042 | 1.010 | 0.970 |

***** OS: overall survival; DFS: disease-free survival.

Note:

1. The risk score function of the 11-gene signature for overall survival = (0.55 × expression level of ITGAV) + (1.08 × expression level of PIK3CA) + (1.15 × expression level of PIK3R3) + (0.92 × expression level of AKT3) + (0.45 × expression level of RAC1) + (0.13 × expression level of ACTN1) + (0.36 × expression level of VCAM1) + (0.75 × expression level of CXCL8) + (0.54 × expression level of CXCL8) + (1.30 × expression level of CTNNB1) + (0.07 × expression level of PTGS2) + (0.01 × expression level of ICAM1).

2. The risk score function of the 11-gene signature for disease-free survival = (0.50 × expression level of ITGAV) + (1.44 × expression level of PIK3CA) + (1.24 × expression level of PIK3R3) + (0.96 × expression level of AKT3) + (0.15 × expression level of RAC1) + (0.78 × expression level of ACTN1) + (0.37 × expression level of VCAM1) + (0.74 × expression level of CXCL8) + (0.52 × expression level of CXCL8) + (0.85 × expression level of CTNNB1) + (0.06 × expression level of PTGS2) + (-0.03 × expression level of ICAM1).

3. The risk score function of the 5-gene signature for overall survival = (1.08 × expression level of PIK3CA) + (1.15 × expression level of PIK3R3) + (0.92 × expression level of AKT3) + (0.75 × expression level of CXCL8) + (0.54 × expression level of CXCL8) + (1.30 × expression level of CTNNB1).

4. The risk score function of the 5-gene signature for disease-free survival = (1.44 × expression level of PIK3CA) + (1.24 × expression level of PIK3R3) + (0.96 × expression level of AKT3) + (0.74 × expression level of CXCL8) + (0.52 × expression level of CXCL8) + (0.85 × expression level of CTNNB1).


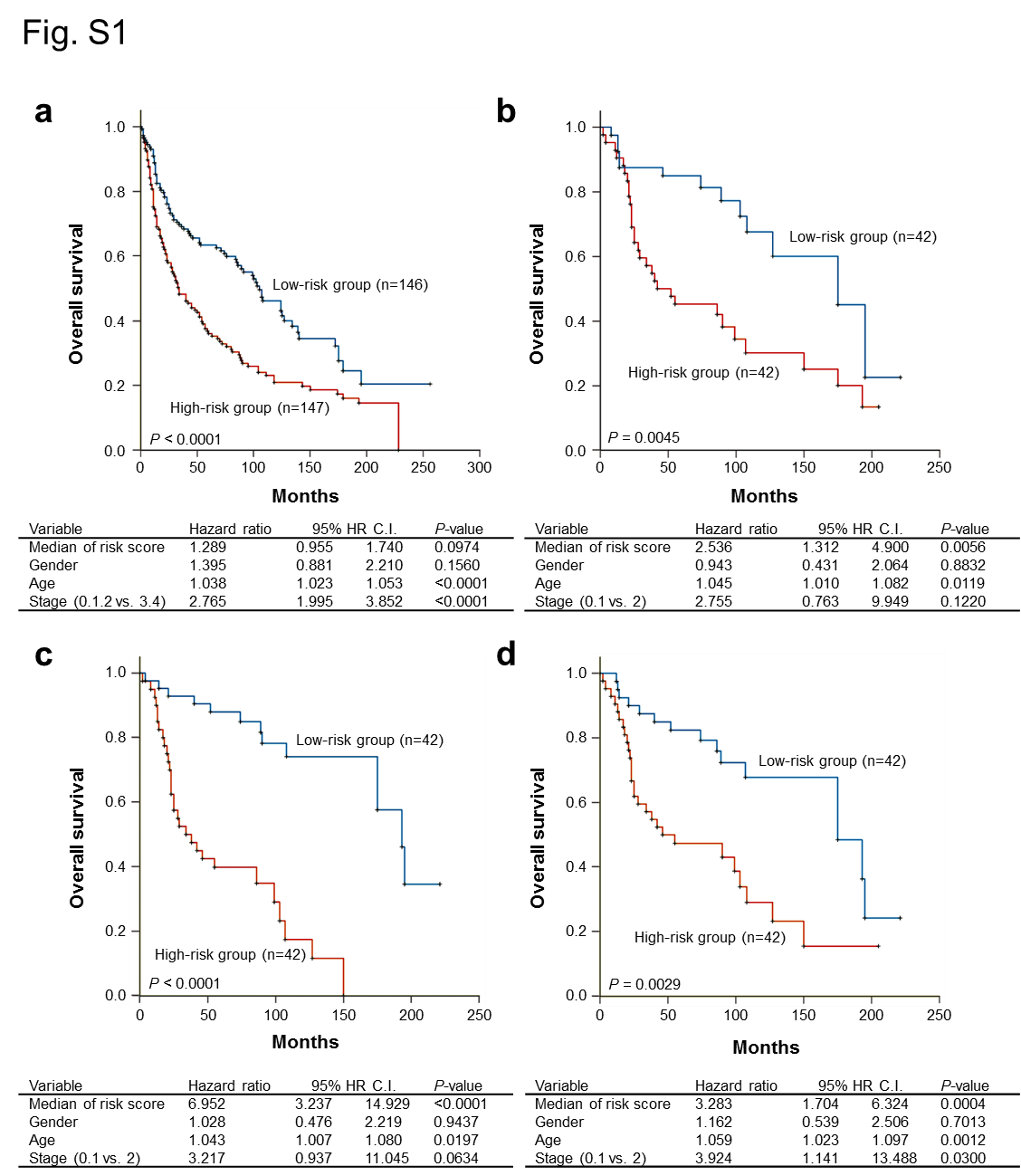


**Fig. S1 Comparison of the endothelial cell derived gene signatures and the published prognostic gene signatures in the Kaplan–Meier estimates of NSCLC patient survival.** (a) Kaplan-Meier estimates of overall survival (n = 293) of the subjects who were categorized based on the 8-gene signature (see ref. 46 in main text). (b) Kaplan-Meier estimates of overall survival (n = 84) of the early-stage lung adenocarcinoma subjects who were categorized based on the 7-gene signature (see ref. 47 in main text). (c, d) Kaplan-Meier estimates of overall survival (n = 84) of the early stage lung adenocarcinoma subjects who were categorized based on the 11-gene (c) and 5-gene (d) signatures derived from the differentially expressed genes of HUVECs co-cultured with cancer cells. The datasets were obtained from GSE30219. The risk score functions of all gene signatures were constructed according to our method as described in this study. The survival curve was estimated by the Kaplan-Meier method, and the log-rank test was performed to test the difference between the survival curves.
